# Man is not a big rat: concerns with traditional human risk assessment of phthalates based on their anti-androgenic effects observed in the rat foetus

**DOI:** 10.1186/2051-4190-24-14

**Published:** 2014-09-02

**Authors:** René Habert, Gabriel Livera, Virginie Rouiller-Fabre

**Affiliations:** Sorbonne Paris Cité, Laboratory of Development of the Gonads, Unit of Stem Cells and Radiation, University Paris Diderot, BP 6, 92265 Fontenay-aux-Roses, France; CEA, DSV, iRCM, SCSR, LDG, 92265 Fontenay-aux-Roses, France; INSERM, Unité 967, F-92265 Fontenay aux Roses, France; Stem Cells and Radiation Unit, LDG / SCSR / iRCM / DSV, Centre CEA, BP6, F-92265 Fontenay aux Roses, France

**Keywords:** Endocrine disruptors, Phthalates, Leydig cells, Masculinization, Human health, Risk assessment, Toxicity test, Development, Reproduction, Foetus, Testis, Testosterone, Perturbateurs endocriniens, Phtalates, Cellules de Leydig, Masculinisation, Santé environnementale, Évaluation du risque, Test de toxicité, Développement, Fœtus, Testicule, Testostérone

## Abstract

Phthalates provide one of the most documented example evidencing how much we must be cautious when using the traditional paradigm based on extrapolation of experimental data from rodent studies for human health risk assessment of endocrine disruptors (EDs). Since foetal testis is known as one of the most sensitive targets of EDs, phthalate risk assessment is routinely based on the capacity of such compounds to decrease testosterone production by the testis or to impair masculinization in the rat during foetal life. In this paper, the well-established inhibiting effects of phthalates of the foetal Leydig cells function in the rat are briefly reviewed. Then, data obtained in humans and other species are carefully analysed. Already in January 2009, using the organotypic culture system named Fetal Testis Assay (FeTA) that we developed, we reported that phthalates might not affect testosterone production in human foetal testes. Several recent experimental studies using xenografts confirm the absence of detectable anti-androgenic effect of phthalates in the human foetal testes. Epidemiological studies led to contradictory results. Altogether, these findings suggest that phthalates effects on foetal Leydig cells are largely species-specific. Consequently, the phthalate threshold doses that disturb foetal steroidogenesis in rat testes and that are presently used to define the acceptable daily intake levels for human health protection must be questioned. This does not mean that phthalates are safe because these compounds have many deleterious effects upon germ cell development that may be common to the different studied species including human. More generally, the identification of common molecular, cellular or/and phenotypic targets in rat and human testes should precede the choice of the toxicological endpoint in rat to accurately assess the safety threshold of any ED in humans.

## Résumé

En toxicologie réglementaire, l’évaluation du risque sanitaire d’un perturbateur endocrinien (PE) est basée sur le paradigme traditionnel qui consiste à extrapoler à l’espèce humaine les données obtenues chez l’animal. Les phtalates fournissent un des exemples les mieux documentés montrant combien nous devons être prudents dans cette démarche. Le testicule fœtal est une cible privilégiée des PE et l’évaluation du risque sanitaire des phtalates a été construite sur la capacité de ces produits à inhiber la production testiculaire de testostérone ou à réduire la masculinisation pendant la vie fœtale chez le rat. Dans cet article, nous présentons brièvement les effets inhibiteurs bien connus des phtalates sur les fonctions des cellules de Leydig fœtales chez le rat. Puis nous détaillons les études effectuées chez l’homme et les autres espèces. Dès janvier 2009, en utilisant un système de culture organotypique original que nous avions mis au point et nommé hFeTA pour human Fetal Testis Assay, nous avons montré que les phtalates ne réduisent pas la production de testostérone par le testicule fœtal humain. En utilisant des modèles de xénogreffes, plusieurs études ont confirmé récemment l’absence d’effet antiandrogénique détectable des phtalates sur le testicule fœtal humain. Les études épidémiologiques ont conduits à des conclusions contradictoires. En définitive, l’effet des phtalates sur les cellules de Leydig fœtales est largement dépendant de l’espèce. En conséquence, on doit s’interroger sur le bien-fondé de l’utilisation actuelle de la dose minimale induisant un déficit de la stéroïdogenèse dans le testicule fœtal de rat pour définir les normes réglementaires d’exposition aux phtalates en santé humaine. Il faut noter que, bien que les phtalates semblent dépourvus d’effet antiandrogénique sur le testicule fœtal humain, ils ne sont pas sans danger puiqu’ils altèrent le développement de la lignée germinale chez l’Homme comme chez toutes les espèces étudiées. De façon plus générale, nous préconisions que l’identification de cibles moléculaires, cellulaires, et/ou phénotypiques communes au rat et à l’homme précéde le choix d’un paramètre critique utilisant le rat comme modèle en toxicologie réglementaire.

## Introduction

The risk of chemicals for human health is routinely assessed by epidemiological and experimental studies. The latters used a traditional risk assessment paradigm based on the integration of both exposure assessment and hazard characterization. Hazard characterization is based on *in vivo* exposure of animals, particularly rats and establishment of a dose response curve allowing the identification of the lowest Non Observed Adverse Effect Level (NOAEL). Then values obtained by this methodology are extrapolated to human to define the Tolerable Daily Intake (TDI). Regulatory Agencies are aware of the difficulties due to the uncertainties linked to extrapolation of experimental data from rodent studies to human health assessment. Therefore, the NOAEL measured in rodent models is divided by a safety factor of 100 to define the regulatory acceptable dose for human health. This safety factor is based on a factor of 10 to account for the differences between rodents and humans multiplied by an additional uncertainty factor of 10 to account for inter-individual human differences in susceptibility.

However, new data show that this rule may be completely inappropriate for Endocrine Disruptor (ED) risk assessment. By comparing the effects of various compounds in rat, mouse and human foetal testes, we have recently demonstrated that the extrapolation to human species of data obtained in rodents is « illogical » in one third of the analyses, whatever the security factor used, because the effect observed in animals does not exist in humans or vice versa [1]. Indeed, whereas fundamental processes in biology, such as apoptosis, cell cycle or cell differentiation, and their pathological disturbances (cancer) are sustained by common basic mechanisms in various species, hormonal regulations are often species-specific in relation with evolution and the large variations in life style. Differences are observed in many hormonal regulatory processes: biochemical nature of the hormone, production level, control of the secretion, metabolism, hormone receptors, signalling molecules… These differences are even more important in reproduction, the most variable function from one species to another. As an example linked with this paper, foetal Leydig cells are stimulated by Chorionic Hormone (CG) as soon as they differentiated in human species whereas there is no CG physiologically active in the rat [2, 3].

Many studies indicate that the incidence of male reproductive function abnormalities in humans is increasing over the years [4–7]. Although differences are observed according to the country and even within regions of the same country, human sperm count has been markedly decreasing over the past four decades. A recent work showed that sperm concentration in France has been declining by 1.9% per year from 1996 to 2005 [8]. Moreover, all studies show that the rate of testicular cancer has been clearly increasing over the last decades. The prevalence rates of cryptorchidism and hypospadias are also probably increasing. According to the most common hypothesis, named the «Testis Dysgenesis Syndrome», these abnormalities result from defaults in the development of the testis during foetal life [9, 10]. Furthermore, many epidemiological, clinical and experimental data suggest that these male reproductive disorders are, at least in part, due to the effects of EDs, which are progressively becoming more concentrated and widespread in our environment [5, 6, 9, 11–20].

ED effects are often more severe when exposure occurs during foetal life than in adulthood [21–26]. Foetal testis thus appears to be a crucial ED target. Accordingly, the tolerable dose intake of a given ED is often derived from the measurement of its effects on the male reproductive functions following foetal exposure. This is the case for phthalates.

It is well established that some phthalates decrease testosterone production by foetal testes in the rat and this anti-androgenic effect is routinely used for human health risk assessment. The aim of this review is to question the validity of this practice because recent data indicate that phthalates do not have detectable anti-androgenic effect in human foetal testes. After a rapid overview of the effects of phthalates on foetal Leydig cell functions and development in the rat, this review will detail data obtained in human foetal testes and finally will extend the analysis to other species.

## Review

### Human exposure to phthalates

Phthalates are phthalic acid esters the use of which has been steadily increasing since 1930. Their worldwide production grew from 1.8 to 4.3 million tons between 1970 and 2006. Phthalates are plasticisers that are added to polymers and principally to PVC to make them softer and more flexible. They are widely used in many soft PVC products, including building and construction materials, such as cabling, flooring, wall covering, profiles and roofs. They are components of medical equipment, hoses, shower curtains, films and plastic gloves, household furnishings, toys, car interiors, clothing, food and beverage packaging, pharmaceutical products, etc. Phthalates are also used as solvents for oil-soluble dyes, insecticides, peroxides and other organic products. They are added to paints and lacquers, adhesives and sealants, cosmetics, lubricants, putty, perfumes, deodorants, sprays. Di(2-ethylhexyl) phthalate (DEHP) was the most widely used phthalate until recently. Now, it is progressively replaced by di-iso-nonyl phtalate (DiNP) which is less biologically active [21].

Phthalates are not covalently bound to the product matrix and can leak out over time. For instance, they are detected in domestic dust [27]. Dermal exposure may also occur via clothes, cosmetics and medical equipment, such as catheters and plastic pockets, for instance. Thus, humans are constantly exposed to phthalates through the oral, dermal and inhalation routes [28, 29]. In the body, phthalates are rapidly hydrolysed by esterases in the gut and other tissues into monoesters that are the active molecules [30, 31]. For example, DEHP is metabolized to its monoester metabolite mono-(2-ethylhexyl) phthalate (MEHP), and di-butyl phthalate (DBP) is converted into mono-butyl phthalate (MBP).

The half-life of phthalates does not exceed 36 h in the body. In humans, 75% of ingested DEHP is metabolized and excreted in the urine within two days [32]. However, phthalates are so widespread in the environment that humans are constantly and largely exposed. According to a study published in 2003, 12% of the German population has a daily DEHP intake that exceeds the European recommendations of 37 micrograms / kg body weight / day [33]. Moreover, all biomonitoring studies highlight the continuous co-exposure to different phthalates. For instance, a North American study estimated that the 95^th^ percentiles of daily exposure to DEHP, DBP, DEP and butylbenzyl phthalate (BBzP) are 9.32, 2.68, 112.3 and 2.47 μg/kg/day, respectively [34]. The concentration of phthalates in human biological fluids shows large individual variations [35–42]. In pregnant women, the mean values in urinary and amniotic fluid samples are 4.10^-7^ M for MBP and 8.10^-8^ M for MEHP. The maximum MEHP concentrations in urinary and amniotic fluid samples are 5.10^-6^ M and 8.10^-6^ M, respectively.

### Effect of phthalates in the rat

A very large body of works demonstrated that some phthalates, those with a C3 to C7 lateral chain such as DEHP, DBP, DiNP, BBzP, di-propyl phthalate (DprP), dipentyl phthalate (DPeP), dihexyl phthalate (DHP), dicyclohexyl phthalate (DCHP), butylbenzyl phthalate (BBzP), and diisoheptyl phthalate (DIHepP), can reduce the functions and development of foetal Leydig cells and consequently impair masculinization in the rat.

#### Disturbances in foetal Leydig cell development and function

In all mammalian species, the first foetal Leydig cells differentiate shortly after the differentiation of seminiferous cords [3, 43–45]. In human testes, the functional differentiation of Leydig cells begins at 6–7 weeks of gestation (GW) and testosterone secretion is highest at around 14 GW and then decreases [46, 47]. In the rat, the first functional Leydig cells differentiate at 15 day post-conception (dpc) and foetal testis steroidogenic activity is highest at around 18.5 dpc (*i.e.,* 3 days before birth) [48]. Then, foetal Leydig cells progressively regress, at least functionally, during the first two weeks of extrauterine life [49–51]. It is important to note that the morphological, functional and molecular characteristics of foetal Leydig cells are different from those of adult Leydig cells, which will differentiate at puberty. For instance, differently from adult cells, foetal Leydig cells are not desensitized by high concentrations of luteinizing hormone (LH)/human chorionic gonadotropin (hCG) [52, 53], their initial differentiation does not depend on LH/hCG [54, 55] and their *in vivo* response to LH/hCG is weak [56]. Furthermore, intratesticular regulations of Leydig cell functions differ between foetus and adult [3]. Moreover, aromatase, a key enzyme in steroidogenesis, is strongly expressed by Sertoli cells in adult testes, while it is essentially expressed by Leydig cells in the foetus [57]. Similarly, FSH, which regulates Leydig cells activity via paracrine factors produced by Sertoli cells, inhibits testosterone production in the foetus and stimulates its production in adult testes [58]. In the rat, exposure to phthalates during the last week of pregnancy induces morphological, molecular and functional alterations in foetal Leydig cells [16, 43, 59, 60]. In response to phthalates, foetal Leydig cells form clusters, develop inside the seminiferous cords and change in size (but not in number) [23, 61–67]. Testis production of testosterone is reduced in a dose-dependent manner due to decreased expression of genes involved in cholesterol metabolism and transport (*Star, HMG-CoA synthase* and *Srb1*) and in testosterone biosynthesis (*Cyp11a*, *3beta-Hsd* and *Cyp17a1*) [68–70]. Recently, Sharpe *et al*. hypothesized that these changes are the consequence of the prevention of Chicken ovalbumin upstream promoter-transcription factor II (COUP-TFII) down-regulation, a competitive inhibitor of steroidogenic factor-1 (SF-1), which is required for the development and maintenance of Leydig cell differentiated function [71].

Foetal Leydig cells also secrete insulin like-3 (INSL-3). This hormone induces the transabdominal descent of foetal testes from their initial mesonephrotic position to the entrance of the inguinal duct, whereas the inguino-scrotal descent is androgen-dependent [72]. Exposure to some phthalates also reduces *Insl-3* expression in the rat foetal testis [73, 74].

A comparison of the effect of various phthalates on testosterone production by rat foetal testes showed that DBP, DEHP, BzBP and diisobutylphtalate (DiBP) are equipotent, dipentyl phthalate (DPP) is about three-fold more potent and diethylphthalate (DEP) has no effect [75].

#### Masculinization defects in the rat

A reduction in foetal Leydig cell function variably impairs masculinization in the rat and these effects are used as endpoints for phthalate risk assessment.

In the male foetus, under the influence of androgens, the Wolffian ducts differentiate into epididimydes, vas deferens and seminal vesicles; the urogenital sinus gives rise to prostate and bulbourethral glands, and scrotum and penis are formed from the urogenital tubercle. In the female foetus, in the absence of androgens, the basic programme of differentiation is maintained: Wolffian ducts regress, while urogenital sinus and tubercle form the lower part of vagina, labia majora and labia minora. Furthermore, the fallopian tubes, uterus and upper part of the vagina spontaneously differentiate from the Müllerian ducts. Conversely in the male, Sertoli cells produce Anti-Müllerian Hormone (AMH) that induces Müllerian duct regression [76].

In the rat, the masculinization/defeminisation of internal and external genitalia begins at 18.5 dpc [77] and therefore many studies on the anti-androgenic effects of phthalates focused on the interval between 18.5 and 21.5 dpc. However, an important period for androgen-dependent masculinization precedes this phenotypic differentiation [78, 79]. Indeed, a reduction of testosterone synthesis or action between 15.5 and 18.5 dpc causes masculinization defects, such as hypospadias, cryptorchidism, incomplete development or agenesis of prostate and seminal vesicles, reduction of the ano-genital distance (AGD) and penis length. It was thus concluded that the development of genitalia is programmed between 15.5 and 18.5 dpc, a period that was named the « masculinization programming window ». A decrease in androgen production after this period does not affect the ontogenesis of male reproductive organs, but reduces their size and the AGD. By analogy with the rat, if a « masculinization programming window » exists in humans, it should be between 7 and 14 GW.

Exposure to phthalates during foetal life causes many masculinization defects in the rat [16, 20, 43, 59, 80–88]. Specifically, phthalates induce hypospadias, cryptorchidism, gubernaculum alterations, defects in differentiation or growth of epididymis, seminal vesicles, deferent ducts, prostate, levator ani and bulbocavernosus muscles, Cowper’s glands, and reduced AGD. Finally, whereas androgens induce nipple regression at 12 days post-partum (dpp) in the rat, phthalates induce nipples retention [89, 90].

All these defects can be easily quantified and are the usual endpoints to establish the NOAEL/lowest observed adverse effect level (LOAEL) of phthalates. For instance, the LOAEL for DEHP as been established to be 11 mg/kg/d based on the reproductive organ malformations in adults after exposure from 8.5 dpc to 17 dpp [91] and 10 mg/kg/d based on AGD measurement at birth and on nipple retention at 12 dpp after exposure from 7.5 to 16.5 dpc [92].

Comparisons of the effect of various phthalates on masculinization showed that DEHP = BBP > DINP>> > DEP, DOTP, DMP [21] and DBP, BBP, DPeP, and DEHP >> > DMP, DEP, and DOTP [93].

### Effect of phthalates in humans

In contrast with the multitude of studies on phthalate anti-androgenic effects in the rat, few data are available on humans [13, 16, 43, 94–96].

#### Epidemiological studies

In 2005, Swann *et al*. found a positive correlation between AGD (measured from the centre of the anus to the anterior base of the penis) and penis volume, testis descent and scrotum volume in 2 to 36 month/old boys in the USA [37]. Therefore, they proposed to use AGD as an easily accessible endpoint to evaluate the androgenic activity of foetal and neonatal testes in humans, similarly to what is done in the rat. They then investigated whether the AGD or the ano-scrotal distance (ASD; measured between the centre of the anus and the posterior basis of the scrotum) in 85 young boys aged from 2 to 36 months was inversely correlated with the concentration of various phthalate metabolites in maternal urine samples collected during the last trimester of gestation. Three years later they strengthened their results by increasing to 106 the number of mother-son pairs [97]. The main result of these two works is the existence of an inverse correlation between AGD and the concentration of MBP, monoisobutyl phthalate (MiBP) and several DEHP metabolites in maternal urine samples. Conversely, AGD was not associated with dibenzyl phtalate (DBzP) or di-*n-*octyl phtalate (DNOP) metabolites. ASD was not associated with any of these phthalate metabolites. Surprisingly both AGD and ASD were associated with monoethyl phtalate (MEP) concentration, a metabolite of DEP, which does not have any anti-androgenic effect in the rat [21, 75]. Finally, the authors observed an inverse correlation between the penis diameter (but not the length) and metabolites of DEHP, but not of other phthalates.

Bustamante-Montes *et al.* [42] confirmed these results [42]. They observed an inverse correlation between MEHP concentration in maternal urinary samples during the last trimester of pregnancy and AGD but not ASD, measured 24-48 h after birth, in 73 mother-son pairs in Mexico State. Moreover, they found a significant inverse correlation between the cumulative MEHP, MBzP, MEP and MBP urinary levels and AGD as well as penis width and stretched length. However, MEHP was undetectable in the urine samples of 24 mothers, while mBzP, MEP and MBP urinary levels were above detection level only in 8–10 mothers.

Choi *et al.* [98] searched for an increase of the concentrations of DEHP, DBP, MEHP, MBP and phthalic acid in the plasma or the urine of 40 children with hypospadias or in the urine of their mother as compared with a control group in Korea [98]. Only DEHP in the urine and phthalic acid in the plasma were increased in the patient boys. Interestingly, bisphenol A plasma concentration was sharply increased (×7) in the plasma of the patient group.

Similarly, Suzuki *et al.* [41] did not find any correlation between AGD at birth and the concentrations of DBP, DMP, DEP, DBzP and DEHP (MEHP and MEOHP) metabolites in maternal urine samples collected between the 9^th^ and the 40^th^ GW in 111 Japanese mother-son pairs [41]. Only AGD, but not ASD, correlation with MEHP concentrations was almost significant.

Lastly, Huang *et al.* [40] did not detect any association between ASD at birth and MEHP or MBP concentration in maternal urine samples collected at 16–20 GW in 33 mother-son pairs in Taiwan [40].

The difficulty to establish an association between exposure to phthalates and androgenic deficiency could be explained by the fact that maternal urine samples are most often collected during the third trimester of pregnancy (*i.e.,* after the masculinization programming window, between 7 and 14 GW). Significant higher risk of hypospadias was found in boys whose mothers were professionally exposed to phthalates (and thus particularly during the first trimester of pregnancy), including hairdressers, beauty therapists, research chemists, line operators, pharmaceutical operators, electrical assemblers, factory assistants, compared to boys whose mothers were not exposed to phthalates at work [99]. However, people who are professionally exposed to phthalates are also exposed to many other confounding anti-androgenic factors.

Another difficulty is linked to the fact that maternal urine levels of a compound or its metabolites may not be indicative of a direct effect of these compounds on the testis in the fetal compartment. This is particularly the case for phtalathes since phthalates are rapidly hydrolyzed by esterases in the gut and other tissues to monoesters, which are the active molecules [30].

The strength of these works could be also limited by the fact that phthalate exposure was always evaluated in a single sample. Nevertheless, according to Teitelbaum *et al.* [100], a single urine sample is often representative of exposure over a 6-month period to warrant its use as an exposure estimate in epidemiological studies [100].

Moreover, phthalates may exert phenotypical anti-androgenic effects by acting on targets different from the fetal testis as suggested by some epidemiological studies that reported an effect also in the female foetuses. Huang *et al.* [40] observed an inverse correlation between the distance from the anus to the posterior convergence of the fourchette in female new-borns and the concentration of MBP in amniotic fluid samples [40]. Furthermore, Sathyanarayana *et al*. [101] reported the association between lower circulating testosterone levels and increased DEHP and DBP exposure in women carrying female foetuses during the second and the third trimester of pregnancy [101]. This association was found also in women carrying male foetuses, but only for DEHP and not DBP.

The main limit of these epidemiological studies is the presence of confounding factors. Specifically, exposure to phthalates is often associated with exposure also to bisphenol A (BPA), a powerful inhibitor of human foetal Leydig cell activity [102]. High levels of plasma BPA have been shown to be strongly associated with hypospadias in Korean boys [98]. The bias resulting from confounding factors could explain the inverse association between AGD and exposure to MEP, a compound without anti-androgenic effects, reported by Swan’s group.

In conclusion, these epidemiological studies do not allow confirming or infirming the existence of an association between phthalate exposure and reduced androgenic activity of foetal testes in humans.

#### Experimental studies

Three independent research groups (headed by K. Boekelheide, R. Habert and R. Sharpe) failed to find any anti-androgenic effect of phthalates in foetal human testes using experimental *in vitro* and *in vivo* models.

**Studies using in vitro models** Historically, the first report on the absence of detection of phthalate anti-androgenic effects in human testes was by the group directed by Richard Sharpe using an organotypic culture model. However, they did not conclude that humans are not responsive to phthalate anti-androgenic effects and attributed this observation to the inability of their *in vitro* system to detect such an effect [63]. Human foetal testes at 15–19 GW were cut in small pieces and the same number of pieces was deposited on filters immersed in culture medium with or without 10^-3^ M MBP for 24 hours. MBP did not change basal, hCG-stimulated and 22R-hydroxycholestorol-stimulated testosterone secretion. As a positive control the authors used rat testes from 19.5 dpc foetuses (a developmental stage comparable with 15–19 GW human foetuses because testis testosterone production is highest at 18.5 dpc in the rat and at 14 GW in humans) [3]. They confirmed that oral gavage in pregnant rats with 500 mg/kg/day DBP at 19.5 dpc reduced testosterone production by foetal testes *in vivo*. Conversely, in their *in vitro* system, 10^-3^ M MBP did not reduce basal and 22R-hydroxycholesterol-stimulated testosterone secretion, but only decreased hCG-stimulated secretion in cultured rat testes. The authors concluded that their *in vitro* approach was not suitable for toxicological purposes and that their observation that phthalates did not alter testosterone production in humans was explained by the limitations of the used *in vitro* method.

**Figure 1:**
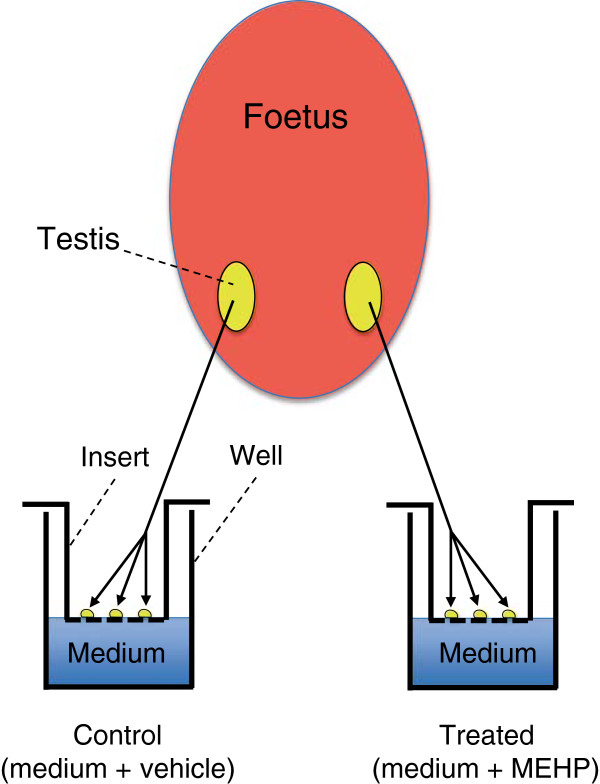
**The Foetal Testis Assay (FeTA) as a usefull tool to measure the effects of Endocrine Disruptors.** Foetal Testis Assay (FeTA) was developed by our laboratory for rat, mouse and human species [47, 103–108, 126]. Human, rat or mouse foetal testes are cut in small pieces of a volume of about 0.2 mm^3^/each piece, at all developmental stages and in all studied species. One to four pieces are randomly placed on Millicell-CM Biopore membranes (pore size 0.4 μm, Millipore, Billerica, MA) floating on 320 μL culture medium in tissue culture dishes. One to eight wells per testis are thus prepared, depending on the species and the age at explantation. The culture medium is phenol red-free Dulbecco modified Eagle medium/Ham F12 (1:1) without addition of any biological factor or hormone. Testes are cultured at 37°C in a humidified atmosphere containing 95% air/5% CO_2_ for three or four days. Medium is completely changed every 24 h. The androgenic and masculinizing activities of cultured testes are evaluated based on the amount of testosterone that is secreted daily by foetal Leydig cells of each testis, on the total number of foetal Leydig cells per testis, or the expression of Leydig cell markers (steroidogenic enzymes or actors, INSL3, LH Receptors).

**Figure 2:**
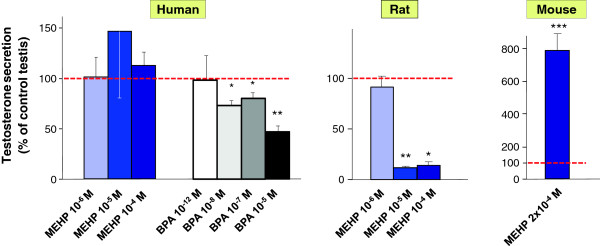
**Comparison of the effects of one phthalate as a function of the species.** Testes at similar stage of development *i.e.* from 7–11 GW human foetuses, 14.5 day-old rat fetuses, or 13.5 day-old mouse foetuses were cultured using the FeTA system described in Figure 1. For each foetus, one testis was cultured in the absence (control) and the other one in the presence of MEHP at concentrations ranging from 10^-6^ to 2×10^-4^ M for 3 days. The daily testosterone secretion was measured by radioimmunoassay. Only values on the third day are presented here but results are similar for the other two days of treatment. Values (means ± SEM) are expressed as the percentage of the secretion of the treated testis compared with that of the control testis. MEHP had negative, positive or no effect with rat, mouse or human fetal testes respectively. Bisphenol A (BPA) at various concentrations was used as positive control for human fetal testes *p < 0.05, **p < 0.01, ***p < 0.001 compared with control testis using the Wilcoxon’s non-parametric paired test. From [1, 102, 109, 121].

Using an organotypic culture system that we developed and named Foetal Testis Assay (FeTA) [16, 47, 103–108] (Figure 1), we affirmed for the first time in January 2009 that phthalates do not reduce testosterone production (and *INSL3* expression) in human foetal testes [109] (Figure 2). We validated this culture system as a powerful *in vitro* model to test the effects of EDs [1, 103, 110].

Testis explants were deposited on an insert at the air-medium interface to allow the survival and development of all testis cell types. Human foetal testes were issued from legal abortions performed between 7–12 GW, a period comparable to 15.5-18 dpc in the rat and corresponding to the « Masculinization Programming Window ». All pieces from one testis were cultured with MEHP concentrations ranging from 10^-6^ M to 10^-4^ M for 3 days. All pieces from the other testis were cultured without MEHP and served as control. Using this system, we showed that, at all concentrations tested, MEHP does not change the daily basal and LH-stimulated testosterone secretion or the expression of genes involved in testosterone biosynthesis (*StAR, CYP11A* and *CYP17A1*). Similarly, also *INSL3* expression was unaffected [103].

Many positive and negative controls validated these conclusions:

- In these testis explants, 10^-4^ M MEHP reduced by 40% the number of gonocytes and by 50% *AMH* mRNA levels. Apoptosis of human gonocytes was significantly increased by exposure to MEHP doses as low as 10^-5^ M [110]. Furthermore, MEHP increased the size of lipid droplets and the expression of LXR alpha, of downstream genes, such as *SREBP1, SREBP2,* and of genes involved in cholesterol and lipid biosynthesis [111].
- Using the FeTA system we showed that human foetal Leydig cells can respond to other EDs as indicated by the finding that exposure to 10^-8^ M BPA reduces testosterone production and *INSL3* expression [102].
- In the FeTA system, exposure to 10^-4^ and 10^-5^ M MEHP reduced the basal testosterone production of rat testes explanted at 14.5 dpc and cultured for three days [1]. Importantly, 14.5 to 17.5 dpc is a developmental period comparable with 7–12 GW in humans. The finding by Hallmark *et al.* [63] that MBP did not have a negative effect on basal testosterone production in foetal rat testes *in vitro* can be explained by the protocol used by these authors who deposited pieces of testes from different animals in the same well [63], whereas in the FeTA system we compare all pieces of one testis to all pieces of the other testis from the same foetus. Moreover, rat foetal testes are less susceptible to phthalates at 19.5 dpc (stage used by Hallmark *et al.*) than at 14.5 dpc. Indeed, addition of 10^-3^ M MBP reduced basal secretion of testosterone by 41% in 19.5 dpc rat testes and by 60% in 14.5 dpc testes cultured in the FeTA system for two days (personal data).
- Other EDs such as diethylstylbestrol reduced testosterone production by the fetal testis in rodents but not in human [11, 102, 112].

In conclusion, although our *in vitro* approach displays intrinsic limits (absence of vascularization, cell survival limited to a few days) it allows the sensitive, direct, rapid, not labour-intensive and relatively cheap evaluation of the toxic effects of a given compound on foetal testis development and functions [1]. Using this method, we never detected any anti-androgenic effect of phthalates in human foetal testes, whereas we always did in rat foetal testes.

**Studies using xenograft models** Three recent studies from the Sharpe’s and Boekelheide’s laboratories described *in vivo* models in which human testes were xenografted in rodents.

Richard Sharpe’s group was the first to develop this approach [113]. Human foetal testes at 14–20 GW were cut in small pieces that were grafted under the skin of castrated CD1 nude mice. In this model, the different testis cell types can survive and develop for six weeks. Specifically, foetal Leydig cell activity (based on the host testosterone concentration or seminal vesicle size) was maintained when the host mice were treated with hCG to mimic normal pregnancy. To evaluate the anti-androgenic effect of phthalates, human foetal testes were xenografted in castrated male nude mice that were then treated with hCG and vehicle, or 500 mg/kg/day of DBP or MBP for 4–21 days [114]. Serum testosterone and seminal vesicle weight did not differ in vehicle-, MBP- and DBP-treated host mice. Conversely, in mice xenografted with 17.5 dpc rat foetal testes (positive control), DBP treatment significantly reduced seminal vesicle weight in the host as well as *Cyp11a1* and *StAR* expression in the graft.

Kim Boekelheide’s group used another approach [115]. Human foetal testes at 10–24 GW were cut in small pieces and grafted in the renal capsule of adult nude male rats that were then force-fed with 0, 100, 250 or 500 mg DBP/kg/day for two or three days. DBP treatment did not change the expression of *INSL3* and key genes in cholesterol metabolism and steroidogenesis (*SCARB1, STAR, CYP11A1 and CYP17A1*) in the grafts. As internal positive control, the authors reported that the number of multinucleated gonocytes in the grafts was increased in response to all DBP doses used. However, they did not observe a reduction in gonocyte density, whereas phthalates decreased gonocyte number by increasing their apoptosis in the FeTA system [109, 110]. As another positive control, the authors grafted 16.5 dpc foetal rat testes in nude rats. Gavage of the host with 500 mg DBP/kg/day for two days reduced by half the ability of the graft to produce testosterone and to express *Cyp17a1, Scarb1* and *Insl3.*

A recent paper from this group confirmed these data [116]. Testis pieces from human foetuses at 10–24 GW were grafted in the renal subcapsular space of castrated adult nude male mice. Animals were then treated with hCG and gavaged with vehicle or 500 mg DBP/kg/day or 75 mg/kg/day of abiraterone acetate (a CYP17A1, inhibitor). The plasma testosterone concentration of the mice was unaffected by DBP, whereas it was dramatically reduced by abiraterone acetate. Furthermore, DBP treatment did not affect the host accessory sex organ weight, whereas the weight of seminal vesicles and levator ani and bulbocavernosus muscle complex was reduced in hosts treated with abiraterone acetate.

In conclusion, although the xenograft model displays intrinsic limits (variability in the survival of grafted pieces, host compensatory reactions, differences between the metabolism in the host and in human…), this approach allows the long-term evaluation of the toxic effects of one chemical compound on foetal testis development and functions. The three mentioned papers used different methods and endpoints, but they all confirmed and extended the results obtained with the FeTA model that phthalates are not anti-androgenic in human foetal testes in experimental settings.

### Effect of phthalates in other species

In stark contrast with the many works on phthalate effects in the rat, very few studies focused on other mammals.

#### Effect of phthalates in the rabbit

In the only available study [22], 400 mg/kg/day of DBP was administered orally to pregnant rabbits from 15 dpc (*i.e.,* just after testis differentiation) to 29 dpc (*i.e.,* three days before birth). DBP treatment decreased serum testosterone levels in male offspring (n = 17) at 6 weeks post-partum, but not thereafter; the weight of testes and accessory sex glands was reduced at 12 weeks post-partum. Furthermore, one rabbit (1/17) had hypospadias, hypoplastic prostate and cryptorchid testes with carcinoma *in situ-*like cells. After reaching adulthood, these rabbits showed a qualitative and quantitative decrease in sperm.

This study suggests that phthalates have anti-androgenic effects in male rabbit foetuses. However, the incidence of reproductive tract malformations following exposure to DBP is lower in rabbits than in rats [43].

#### Effect of phthalates in the mouse

The first study was performed by Boekelheide’s group [117]. Gavage of pregnant mice with DBP (1500 mg/kg/day), MBP (1000 mg/kg/day) or MEHP (1000 mg/kg/day) from 14.5 to 16.5 dpc or from 15.5 to 17.5 dpc did not change foetal testis testosterone concentration and *Scarb1* and *Cyp11a1* mRNA expression at 17.5 dpc. Importantly, like in the rat, these treatments caused an increase in multinucleate gonocytes. Gavage with an unique dose of DBP (500 mg/kg) at 18.5 dpc did not reduce the expression of key genes involved in cholesterol transport and metabolism (*Scarb1, Dhcr7, Star*) and in testosterone biosynthesis (*Cyp11a1, Cyp17a1*) measured 4–8 h after gavage. These genes were all down-regulated in rat foetuses treated using a similar protocol [68]. The authors concluded that, differently from the rat, phthalates do not have an anti-androgenic effect in mouse foetal testes.

On the other hand, Liu *et al.* [118] observed a dose-dependent increase in hypospadias in new-born mice from pregnant animals that received 100, 200 or 500 mg/kg/day of DEHP from 12.5 dpc (*i.e.,* when the first foetal Leydig cells differentiate) to 17.5 dpc (*i.e.,* two days before birth) [118]. AGD was also decreased in animals exposed to the highest dose.

Do *et al.* [119] gave 0, 0.0005, 0.001, 0.005, 0.5, 50 or 500 mg/kg/day of DEHP to pregnant mice from 9 to 18.5 dpc [119]. With the exception of the 500 mg/kg/day dose that did not have any effect, plasma and testis testosterone levels and AGD measured 2–4 hours after the last gavage were increased in male foetuses according to an inversed U-shaped dose–response curve in agreement with the results by Gaido *et al.* [117].

In another study, 100, 200 or 500 mg/kg/day DEHP given to mice from 12.5 dpc to 3 dpp decreased testis *Insl3* expression measured at 5 dpp in a dose-dependent manner [120].

Heger et al. [115] analyzed mouse testes from 15.5 dpc mouse implanted into adult male immunodeficient host rats, which were then given for 2 days to 0, 250 or 500 mg/kg/day DBP by oral gavage [115]. Quantitative RT-PCR analysis did not reveal any change of the expression of the Leydig cell–specific genes *Ex vivo* testicular incubation showed slight DBP-induced increase in testosterone production. On the opposite, all these parameters were decreased by DBP in 16.5 dpc rat grafted fetal testes.

Finally, an *in vitro* study highlighted the complexity of the response to phthalates in the mouse and tried to reconcile the contradictory data described above [121]. Mouse foetal testes were cultured using the FeTA system in the presence or not of 2.10^-4^ M MEHP for three days. MEHP increased basal testosterone production by testes explanted at 13.5 or 18.5 dpc, but decreased LH-stimulated testosterone secretion by testes explanted at 18.5 dpc. As LH secretion physiologically appears in late foetal life, these *in vitro* data explain why phthalates have antiandrogenic effects in the mouse only during late foetal or neonatal life, while they do not have any effect or show positive effects before late foetal life in the *in vivo* experiments described above.

#### Effect of phthalates in the marmoset

The group headed by Richard Sharpe tested the effects of phthalates also in marmosets. Indeed, this non-human primate (*Callithrix jacchus*) could be a better model for assessing the effects on humans than rodents [122]. Pregnant female marmosets received 500 mg/kg/day of MBP from the 7^th^ to 15^th^ week of gestation and male offspring were studied at birth. This period overlaps with the human «masculinization programming window» because the formation of the gonad occurs during week 6 and the duration of pregnancy is of 21 weeks in this species [123]. MBP did not affect gross testicular morphology, reproductive tract development or testosterone levels at birth. Conversely, a similar treatment in rats causes 17% hypospadias and 70% cryptorchidism [64]. Differently from what observed in human and rat testes, neither the number nor the differentiation of Sertoli cells and gonocytes was affected by MBP. Multinucleated gonocytes were not observed in both control and MBP-treated new-born marmosets, but abnormally aggregated gonocytes were detected in 33% of treated new-borns, as previously reported in the rat [124].

Although this study did not include positive controls and did not demonstrate that with this protocol DBP/MBP could reach the foetal testes, it suggests that Leydig cells are not affected by phthalates during foetal life in marmosets.

Table 1 schematically summarizes the differences observed in the various studied mammals.

**Table 1:** **Effect of DEHP/MEHP and DBP/MBP on fetal Leydig cell functions in human and various species**

	Species	Stage of development of the fetal testis at exposure	Observation	References
Epidemiological studies	Human		Evidence for an association between a reduction of masculinization and phthalates exposure	[37], [42, 97, 99]
			Weak evidence for an association between a reduction of masculinization and phthalates exposure	[41], [98, 101]
			Not any association between masculinization and phhtalates exposure	[40]
Experimental studies	Human	Early stages	No effect of phthalates	[109], [110]
		Late stage	No effect of phthalates	[63], [114–116]
	Rat	Early stages	Strong negative effect of phthalates	[1], [23, 25, 61, 63–70, 75, 80–88, 90–93]
		Late stages	Negative effect of phthalates	[63], [89]
	Mouse	Early stages	Positive effect of phthalates	[115], [118, 119, 121]
		Late stages	Negative or no effect of phthalates	[117], [121]
	Rabbit	Early + late stages	Negative effect of phthalates	[22]
	Marmoset	Early + late stages	No effect of phthalates	[122]

## Conclusion

The toxicological reference values of various phthalates are mostly based on their anti-androgenic effects in the rat foetus; however, more and more studies show suggest that these effects do not exist in humans. Similarly, phthalates do not reduce foetal Leydig cell activity in the mouse during the beginning of testis development and in foetal marmoset, whereas they might have a weak anti-androgenic effect in rabbits. This suggests that phthalates suppress foetal Leydig cell steroidogenesis specifically in the rat.

The explanation for this species difference is not yet established. However, Veeramachaneni and Klinefelter recently observed that, DEHP exposure induced an increase in circulating oestradiol in the rat and they supposed that aromatase is one of the primary targets of phthalates [96]. Since oestradiol reduces testosterone production by the rat fetal testis [112] but not by the human fetal testis during the first or the second week of pregnancy [102, 125] this could explain the species difference concerning phthalate anti-androgenic effects. The species difference in the oestrogen anti-androgenic effect has been explained as follow : estradiol reduced fetal testicular testosterone production *via ESR1* [11, 102, 126] and ESR1 expression (mRNA and protein) is not detected in human fetal testes [127].

This review focused only on phthalate exposure during foetal life, because this period is particularly sensitive to endocrine disruption and, thus, intrauterine exposure to phthalates is normally used to evaluate phthalate hazards. The critical susceptibility to EDs extends to neonatal life, but no experimental data on the adverse effects of phthalates in humans during this developmental period are presently available. An anti-androgenic effect of phthalates during this period cannot be excluded because foetal and neonatal Leydig cells are different in human [3] and exposure to DBP reduces plasma testosterone levels in marmoset neonates, but not in foetuses [63].

While phthalate effects on Leydig cell development and function are highly variable from one species to another, their adverse effect on the seminiferous compartment has been reported in foetuses of all studied species. However, it is difficult to perform a precise inter-species comparison in this case because the number of studies is very limited. Furthermore the analysed endpoints differ from one study to another. It is clearly established that phthalates increase the development of multinucleated gonocytes in the rat during late foetal life [23, 64, 124, 128, 129] and in the mouse [117, 130]. However, this was not observed in marmosets [122], and in humans phthalate-induced multinucleated gonocytes were not detected during the first trimester of gestation [109] but only later, starting from the second trimester [115, 116]. Gonocyte apoptosis may be another phthalate-responsive endpoint common to different species because it was observed *in vitro* [131] and *in vivo* [129] in the rat, and *in vitro* in mouse [121] and human foetal testes [103, 110]. Unfortunately, this endpoint has not been studied in xenografted human foetal testes. It is not clear whether phthalate-induced disturbances in gonocyte development result from a direct effect on this cell type or whether they are mediated by Sertoli cells. Indeed, Sertoli cell proliferation and/or gene expression are altered by phthalates both in rat and human foetuses [103, 132, 133]. In conclusion, as gonocytes and Sertoli cells are common targets of phthalates in different species, including humans, efforts should be made to define a set of genes the expression of which is changed by phthalates both in rat and human testes in order to establish new endpoints for human risk assessment.

More generally, the identification of common molecular, cellular or/and phenotypic targets in both rat and human models should precede the choice of a toxicological endpoint in the rat to accurately assess the safety threshold of any ED in humans.
